# Hepatic tuberculosis presenting with extreme hyperferritinemia masquerading as adult-onset Still’s disease: a case report

**DOI:** 10.1186/1752-1947-6-195

**Published:** 2012-07-12

**Authors:** Edirisooriya Maddumage Manoj, Rajini Srigrishna, Murugapillai K Ragunathan

**Affiliations:** 1 Ward 45, National Hospital of Sri Lanka, Colombo, Sri Lanka

**Keywords:** Ferritin, Adult-onset Still’s disease, Tuberculosis, Granulomatous hepatitis

## Abstract

**Introduction:**

Isolated hepatic tuberculosis is an uncommon manifestation of one of the most common infections worldwide, caused by *Mycobacterium tuberculosis.* Extremely high serum ferritin, which is regarded as a marker of adult onset Still’s disease, has not been observed in patients with tuberculosis of the liver. We report a case of hepatic tuberculosis who presented with clinical criteria of adult-onset Still’s disease and extreme hyperferritinemia, which posed a diagnostic confusion.

**Case presentation:**

Our patient was a 48-year-old Sri Lankan man who presented with fever, polyarthralgia and a generalized skin rash of three months duration. He had marked constitutional symptoms, oral ulcers, hair loss, anemia and hepatomegaly. Laboratory investigations disclosed an inflammatory syndrome, evidence of hepatic dysfunction, bone marrow suppression and a raised serum ferritin level of 34,674 ng/ml. A rapidly deteriorating course of illness prompted treatment based on a presumptive diagnosis of adult-onset Still’s disease until liver histology was available. The patient died of sepsis followed by multi-organ dysfunction. Later, the liver histology revealed tuberculosis.

**Conclusion:**

Extrapulmonary tuberculosis, although well known to present with peculiar manifestations, has not been reported to be associated with extremely high levels of serum ferritin in immunocompetent individuals. Isolated hepatic tuberculosis presenting with clinical criteria of adult-onset Still’s disease is remarkable. Since tuberculosis remains a potentially curable disease, an awareness of its’ protean manifestations is essential.

## Introduction

Adult-onset Still’s disease (AOSD) is a rare systemic inflammatory disorder of uncertain etiology. Several micro-organisms, especially viruses, have been postulated in the pathogenesis of juvenile and AOSD [1]. Tuberculosis (TB) is considered a common devastating infection which may affect any organ of the body giving rise to peculiar clinical presentations. Often this may lead to a considerable confusion in diagnosis unless there is a strong suspicion of mycobacterial infection. Although TB affecting the liver is not a rare entity, isolated hepatic TB presenting as fever, skin rash and joint pains with extremely high serum ferritin is remarkable. To the best of our knowledge, hepatic TB presenting with clinical criteria of AOSD has not been reported previously.

## Case presentation

We report the case of a 48-year-old Sri Lankan man who was admitted to our facility with an intermittent fever associated with joint pains and a skin rash for three months. He had an inflammatory type symmetrical arthralgia confined to large joints with early morning stiffness for 30 minutes. Skin rash, which was non-itchy, non-scaly and non-photosensitive, appeared initially on the trunk but became generalized within a period of two weeks. He was overwhelmed with marked malaise, severe anorexia, weight loss of 19 kg over three months with significant hair loss and multiple painful oral ulcers. He had a watery diarrhea of three to four bowel movements a day of one month duration associated with occasional episodes of vomiting and a vague abdominal pain. His past medical history was unremarkable except for two uncomplicated episodes of malaria about twenty years previously. He denied either exposure to high risk sexual activities or intravenous drug use. He did not consume alcohol and was a non-smoker.

**Figure 1:**
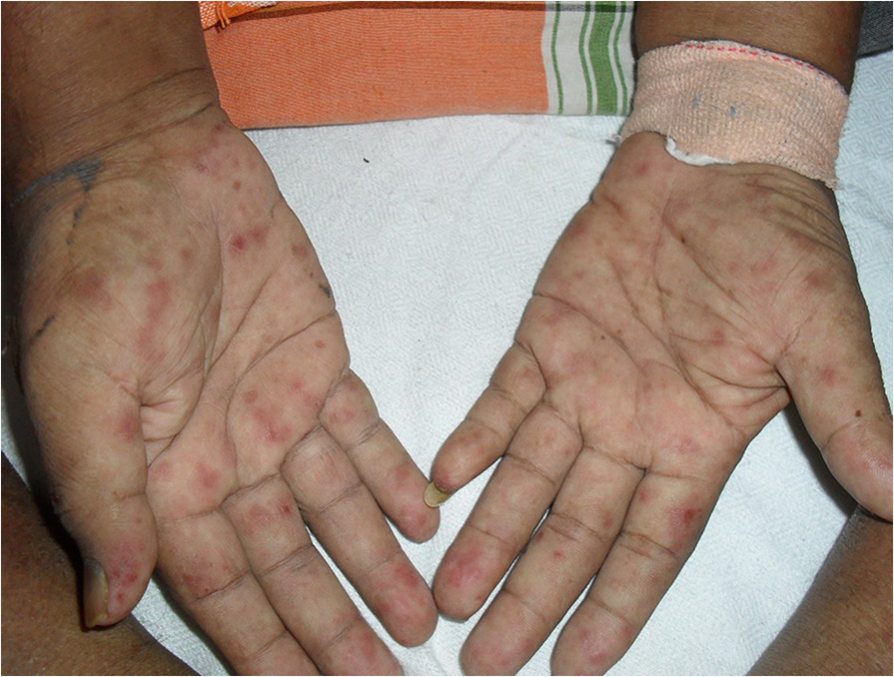
Generalized erythematous rash extending to palms and soles.

On examination, the patient was ill looking, febrile (temperature of 38.4°C), dyspneic and moderately pale. Jaundice, finger clubbing, cyanosis and lymphadenopathy were absent. He had a generalized erythematous maculopapular skin rash involving the palms and soles (Figure 1), a non-scaring alopecia and multiple shallow ulcers in the oral mucosa. On admission, his pulse rate was 100/minute, his blood pressure was 100/70 mmHg and his respiratory rate was 46/minute. Breath sounds were reduced in the base of the right lung with occasional rhonchi heard over both lung fields. He had a tender, firm hepatomegaly of 14cm span with a smooth surface and a regular edge. There was no splenomegaly, abdominal masses or ascites. His rectal examination was unremarkable but several non-painful ulcers were present in his scrotum. Optic fundus showed cotton wool spots around the disc; otherwise his neurological examination was normal. Deformities or edema of the small or large joints were absent but joint line tenderness was demonstrable in his wrist, elbow, shoulder, knee, and ankle joints bilaterally. However, his spinal movements were preserved and movements of the pelvic and shoulder girdles were painful at full range.

**Figure 2:**
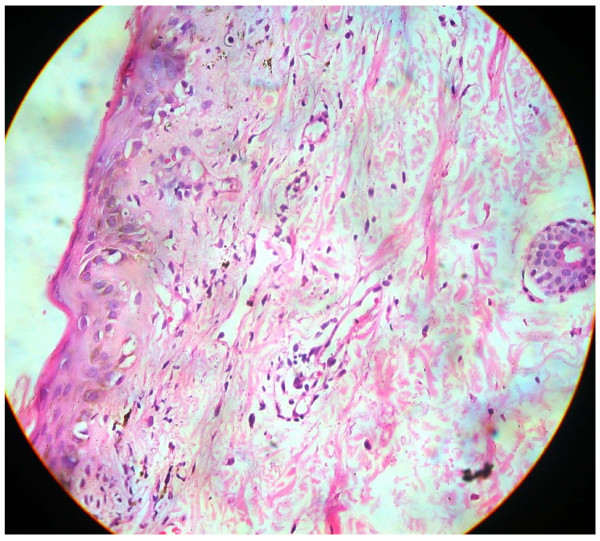
**Skin biopsy (10 x 40). Epidermis is thinned out with extensive vacuolar degeneration of basal cells with focal spongiosis.** There is pigment incontinence and moderate mononuclear cell infiltrates in the papillary dermis. The dermis is unremarkable. There is no evidence of vasculitis, edema or mucus deposition.

Laboratory tests revealed normochromic normocytic anemia (hemoglobin: 8.7g/dL, mean cell volume: 86.5fL) with a total leucocytes of 5.4 x 10^9^/L (neutrophils: 75%, lymphocytes: 19%), and a platelet count of 64 x 10^9^/L. Inflammatory markers were elevated (erythrocyte sedimentation rate: 140mm; C-reactive protein: 60mg/dL) and he had an extremely high serum ferritin level of 34,674ng/ml. Arterial blood gas analysis revealed moderate hypoxia (PaO_2:_ 78.7mmHg). Chest X-ray did not show evidence of consolidation or cavitations but his right hemidiaphragm was elevated. His liver functions were abnormal (aspartate aminotransferase: 252IU/L [<35IU/L], alanine aminotransferase: 69IU/L [<35IU/L], alkaline phosphatase: 1857IU/L [<300IU/L], gamma glutamyl-transferase: 2089IU/L [<54IU/L]) with a reversed albumin/globulin ratio (albumin: 1.9g/dL, globulin: 4.6g/dL). Serum billirubin (11.9μmol/L) was not elevated and serum creatinine (1mg/dL) was normal. Abdominal sonography revealed uniformly increased echogenicity of the enlarged liver but evidence of billiary obstruction or focal lesions was absent. A battery of serological investigations including enzyme-linked immunosorbent assay (ELISA) for human immunodeficiency virus (HIV), a hepatitis panel, blood testing for malaria parasites, rickettsial antibody, sputum for acid fast bacilli acid-fast bacillus acid-fast bacillus (AFB) and tuberculin skin test were all negative. Repeated blood cultures did not yield any bacterial growth and a venereal disease research laboratory screen for syphilis (VDRL) was non-reactive. His rheumatoid factor was 128IU/mL(<8IU/mL) but autoantibody screen including anti-nuclear factor, U1 ribonucleoprotein (RNP) complex, anti-smooth muscle antibody (ASMA), antimitochondrial antibody (AMA) and anti-neutrophil cytoplasmic antibodies (ANCA) were negative. Biopsy of the skin rash was inconclusive (Figure 2). Bone marrow examination revealed evolving marrow hypoplasia although evidence of marrow infiltration by metastatic deposits or granulomata was absent. Polymerase chain reaction (PCR) for *Mycobacterium tuberculosis* in bone marrow aspirates was negative. His clotting profile was mildly abnormal with a prothrombin time of 15 seconds (INR: 1.3) and a partial thromboplastin time of 58 seconds (control 35 to 42 seconds).

His dyspnea and tachypnea, attributable to bronchospasm associated with systemic inflammatory reaction (SIRS) was probably worsened by high fever, marked myalgia, right hypochondrial tenderness with enlarged liver and anemia. Considering the possibility of sepsis, broad spectrum antibiotic treatment with intravenous meropenem 1g daily and metronidazole 500mg every eight hourly was instituted from the day of admission. His symptomatology and respiratory distress was only slightly improved with bed rest in a propped up position, antipyretics, salbutamol nebulization, analgesics and blood transfusions to correct the anemia. He underwent liver biopsy on day two of admission after the correction of clotting abnormalities.

**Figure 3:**
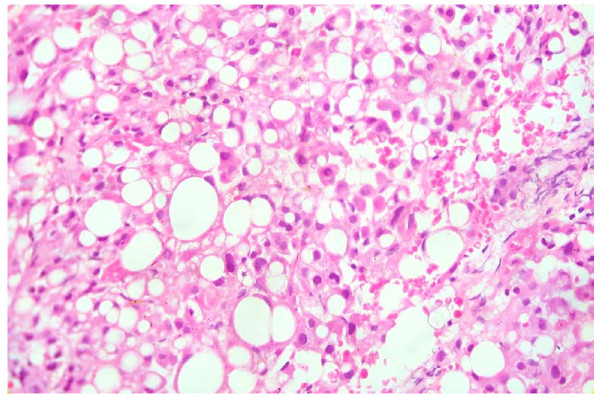
** Liver histology (10 x 40); Noncaseating granuloma in the background of a fatty liver.** No portal tracts were included in the biopsy. There is no evidence of acute inflammation, cirrhosis, dysplasia or malignancy.

A presumptive diagnosis of AOSD with granulomatous liver disease was made based on the clinical criteria of Yamaguchi (Table 1) and extremely high serum ferritin levels [2,3]. He was started initially on indomethacin 25 mg every eight hourly followed by intravenous methylprednisolone 1g daily pulses for three days under broad spectrum antibiotic cover. These measures failed to improve his clinical picture. He died on the sixth day after admission due to sepsis and multi-organ dysfunction. The liver biopsy revealed noncaseating granulomas in the background of fatty liver, later found to be culture positive for *M. tuberculosis* (Figure 3).

**Table 1:** Yamaguchi criteria

**Major criteria**	
·	Fever ≥39°C (one week or longer)
·	Arthralgia and/or arthritis (two weeks or longer)
·	Non-pruritic, pink, macular or maculopapular rash, usually during febrile episodes (evanescent, salmon-pink rash)
·	Leucocytosis (>10,000mmol/L, >80% neutrophils)
**Minor criteria**	
·	Pharyngitis
·	Lymphadenopathy and/or splenomegaly
·	Liver involvement (raised serum transaminases and/or lactate dehydrogenase)
·	Negative rheumatoid factors and antinuclear antibodies

Diagnosis of adult-onset Still's disease needs five or more of the diagnostic criteria listed above, of which two must be major criteria.

## Discussion

We describe the case of a previously immunocompetent man presenting with clinical criteria of AOSD with extremely high serum ferritin, secondary to hepatic TB. In extra pulmonary TB, hepatic involvement has been regarded as uncommon but not an exceptional manifestation [4]. Most of the cases usually occur in association with milliary TB, mainly acquired through hematogenous dissemination. Our patient did not have convincing evidence of pulmonary or direct bone marrow involvement to suggest disseminated TB. Ultrasound examination and computed tomography (CT) findings have a low specificity in the diagnosis of liver involvement in TB [5].

Ferritin is a high molecular weight iron-containing protein that functions as an iron storage compound. Under normal circumstances, the amount of ferritin synthesized and secreted into the serum is proportional to the quantity of body iron stores. However, serum ferritin concentrations may be elevated out of proportion to iron stores in the presence of certain clinical syndromes, including liver disease [6], renal disease [7], HIV infection [8], non-HIV systemic infections or inflammation [9], malignancies [9-11] chronic red-cell transfusion [12,13] and sickle cell syndrome [13]. Extremely high level of ferritin (>10,000ng/ml) is sometimes suggested as a marker in the diagnosis of AOSD [14,15]. An extensive literature search showed that extreme hyperferritinemia is rarely reported except in patients with AOSD (Table 2). There are no pathognomonic clinical or laboratory findings for AOSD. Therefore, it is still a diagnosis of exclusion. Serum ferritin levels of >3000ng/mL have been most commonly observed and, particularly, the glycosylated ferritin level drops to ≤20% in patients with AOSD [16]. Out of eight different sets of criteria to diagnose AOSD, the most widely used and validated criteria are those of Yamaguchi (Table 1). The diagnosis of AOSD requires five or more of the criteria, of which two must be major criteria with a sensitivity of 96.2% and specificity of 92.1%. Our patient had three major and two minor criteria compatible with the diagnosis of AOSD. The exclusion of other potential diagnoses such as infections (notably TB, toxoplasmosis, infectious mononucleosis, deep abscesses, and so on), connective tissue disorders, neoplasms as well as certain drug reactions is a key step in the diagnosis of AOSD. Although this elimination process appears long, the delay may be necessary to avoid therapeutic errors.

**Table 2:** Literature review of cases with extremely high serum ferritin

**Ref.**	**Sex/Age**	**Clinical features**	**Labs**	**Ferritin**	**Diagnosis**
				**(ng/ml)**	
[17]	M/23y	Fever	WBC 5800/ mm3	13,547	HIV with milliary TB
		Asthenia			
		Malaise			
[18]	F/40d	Fever	WBC 2400/ mm3	25,534	Hemophagocytic lymphohistiocytosis
		Lymphadenopathy			
		Hepatosplenomegaly			
[19]	M/77y	Cardiac arrest	ALT; 4410IU/L	10,740	Acute hepatic damage
[19]	M/75y	Fever, hypotension	ALT;2530 IU/L	46,500	Septicemia
			Iron: 12umol/L Tr.: 40%		
[19]	F/64y	Liver transplant	ALT;5790IU/L	42,510	Post-operative Hepatic ischemia
			Iron: 49 umol/L Tr. : 79%		
[19]	F /59y	Liver transplant	ALT;1790IU/L Iron 26 umol/L Tr.t: 45%	15,100	Post-operative hepatic ischemia
[19]	F/61y	Cardiac catheterization	ALT : 2510IU/L	23,200	Dissection of coronary arteries and hypotension
[20]	F/49y	PUO Hepatosplenomegaly Pleural effusion Rash;erythema-multiform	Leucocytosis DIC Hemophagositic-syndrome	240,000	Breast carcinoma Paraneoplastic syndrome

*ALT* alanine aminotransferase, *DIC* disseminated intravascular coagulation; *F* female; *M* male, *PUO* pyrexia of unknown origin, *Ref* reference, *Tr* transferrin saturation, *WBC* total white blood cells.

Evolving bone marrow hypoplasia and granulomatous hepatitis can both sometimes be associated with AOSD. Bone marrow involvement in AOSD is explained as hemophagocytic syndrome and is considered a poor prognostic sign [21]. Hemophagocytic syndrome, defined as phagocytosis by macrophages of erythrocytes, leukocytes, platelets, and their precursors in bone marrow and other tissues, is an unspecific phenomenon found in several conditions such as hemolytic anemia, malignant disease and infections. In our patient, although bone marrow hypoplasia was present, convincing evidence for hemophagocytic syndrome was not seen.

The liver biochemistry of our patient can be interpreted as infiltrative disease favoring granulomatous hepatitis. Granulomatous hepatitis can be associated with autoimmune or rheumatological conditions, malignancies, systemic infections or drugs, but the most common cause is TB [22]. However, if a patient presents more atypically, as did our patient, clinicians may initially not suspect TB as the diagnosis. Absolute exclusion of TB is a huge challenge in a resource limited setting such as Sri Lanka. The variable sensitivity and reliability of certain tests, such as tuberculin skin test, sputum for acid-fast bacillus acid-fast bacillus (AFB), TB-polymerase chain reaction (PCR), and so on. is a considerable barrier to confident early exclusion of TB. In our case, the decision making process was further compromised by the late presentation of our patient and the considerably long time needed for the gold standard test, *M. tuberculosis* culture to confidently exclude TB.

Unfortunately, our patient, who presented late in the course of deteriorating illness, prompted us to make an early therapeutic decision to entertain the presumptive diagnosis of AOSD. A trial of anti-tuberculous therapy (ATT) was precluded by well known liver and bone marrow toxicity effects of ATT in a patient with already impaired hepatic and hematopoetic functions in the background of a lack of solid evidence of TB. The retrospective analysis of this case would justify a trial of ATT or to combine ATT with parenteral steroids in our patient considering the dramatic consequences. Delayed histological diagnosis due to late presentation, atypical manifestations, poor prognostic features such as bone marrow involvement and immunosuppression with corticosteroids would eventually have accelerated the deterioration of his condition.

## Conclusions

This patient gives us a caution that exclusion of TB is a high priority in differentiating causes of possible granulomatous liver disease. This step is pivotal, especially when the immunosuppressive therapy is proposed for patients living in a country that is hyperendemic for *M. tuberculosis*. On the other hand, this is probably the first reported case of hepatic TB presenting with extremely high serum ferritin levels and clinical criteria of AOSD. Since TB remains a potentially curable disease, an awareness of its protean manifestations is essential.

## Consent

Written informed consent was obtained from the patient’s next-of-kin for publication of this case report and accompanying images. A copy of the written consent is available for review by the Editor-in-Chief of this journal.

## Competing interests

The authors declare that they have no competing interests.

## Authors’ contributions

EMM analyzed and interpreted the patient data and uncommon presentation of TB infections. The literature review was done by RS. MKR guided the others for reporting this case and corrected the final manuscript. All authors read and approved the final manuscript.
